# Report of an Extraordinary Double Birth

**Published:** 1848-11

**Authors:** W. F. Askham

**Affiliations:** Surgeon, Eckington, Derbyshire


*Report of an Extraordinary Double Birth.* By W. F. Askham, Esq., Surgeon, Eckington, Derbyshire.—I was called upon, on the 3d of February last, to attend a healthy young woman, then in labour of her fourth child, and according to her own statement, at the full period of utero-gestation. As there had been hæmorrhage to some extent previous to my arrival, I at once made an examination, and found the presentation a natural one, and the labour so far advanced, that nothing but an active pain or two appeared wanting to complete it. These soon came on, and the head was expelled, and almost immediately afterwards the shoulders, without difficulty. During the passage of the breech, however, I discovered something unusual ; but before I could satisfy myself what it was, another vigorous contraction of the uterus took place, and the whole was expelled.

I was surprised to see a *lusus naturæ* of a very extraordinary character—two apparently well formed, but still born children, united from the sternum to the lower part of the abdomen, or rather, to the umbilicus—the one a male, but the other without any external organs of generation, or anus. When laid on a table, they lie nearly face to face. Examined internally, the following appearances presented themselves :—

Each child has its separate spine, one of which is considerably distorted by lateral curvature. There are two sterna, one forming the anterior, and the other the posterior boundary of the cavity which contains the thoracic viscera of both bodies, and is divided, vertically, by a fibro-tendinous membrane. One of the hearts—for there are two—is as large as a pullet's egg ; but the other no larger than a hazel nut. Each body has its set of lungs. The œsophagus of each passes into the stomach proper to it. Both duodena are natural at their origin, but soon unite and form one canal ; and at their junction they receive the ductus communis choledochus. This common intestine is continued for about two feet, when it expands into a triangular-shaped sac, of considerable size, from each of the inferior angles of which a small intestine issues, that on the left side continuous with a colon, cæcum, and rectum; but the other terminating shortly in a pouch, without any outlet that can be discovered. The abdominal cavity is common to both bodies. The liver is divided into two large lobes, each having a gall-bladder and duct; and the ducts unite before entering the intestine, at the point where the duodena join, and form one canal. The kidneys of the left child are large; those of the other, small. The bladder of each is normal. There is one placenta, and one funis; and the latter, at its point of insertion into the umbilicus, is of very considerable size. The right child has the imperforate anus, and the small sized heart and kidneys; and it had also on one hand an extra thumb. In other respects it much resembles the other, and is of equal size and development. The smaller heart is perfect in structure, the auricles and ventricles being quite distinct; and the different vessels arising from it are as large as those arising from the more fully developed heart of the other child. The blood vessels have not been further examined, from an unwillingness to injure the preparation. I may add, that notwithstanding it is the woman's opinion she had attained to the full period of pregnancy, my own is, that she was three weeks or a month short of it at the time of her labour.—*Lancet.*

